# The European Bioinformatics Institute in 2020: building a global infrastructure of interconnected data resources for the life sciences

**DOI:** 10.1093/nar/gkz1033

**Published:** 2019-11-08

**Authors:** Charles E Cook, Oana Stroe, Guy Cochrane, Ewan Birney, Rolf Apweiler

**Affiliations:** European Molecular Biology Laboratory, European Bioinformatics Institute (EMBL-EBI), Wellcome Genome Campus, Hinxton, Cambridge CB10 1SD, UK

## Abstract

Data resources at the European Bioinformatics Institute (EMBL-EBI, https://www.ebi.ac.uk/) archive, organize and provide added-value analysis of research data produced around the world. This year's update for EMBL-EBI focuses on data exchanges among resources, both within the institute and with a wider global infrastructure. Within EMBL-EBI, data resources exchange data through a rich network of data flows mediated by automated systems. This network ensures that users are served with as much information as possible from any search and any starting point within EMBL-EBI’s websites. EMBL-EBI data resources also exchange data with hundreds of other data resources worldwide and collectively are a key component of a global infrastructure of interconnected life sciences data resources. We also describe the BioImage Archive, a deposition database for raw images derived from primary research that will supply data for future knowledgebases that will add value through curation of primary image data. We also report a new release of the PRIDE database with an improved technical infrastructure, a new API, a new webpage, and improved data exchange with UniProt and Expression Atlas. Training is a core mission of EMBL-EBI and in 2018 our training team served more users, both in-person and through web-based programmes, than ever before.

## INTRODUCTION: ARCHIVAL RESOURCES AND KNOWLEDGEBASES

EMBL-EBI data resources cover the entire range of molecular biology and include nucleotide sequence data, protein sequences and families, chemical biology, structural biology, systems, pathways, ontologies and the scientific literature. EMBL-EBI’s data resources collate, integrate, curate and make freely available to the public the world's scientific data.

Our resources (www.ebi.ac.uk/services) include archival or deposition databases that store primary experimental data submitted by researchers, as well as knowledgebases that integrate and add value to experimental data, with many having both functions (1,2). All EMBL-EBI data resources, are open access and freely available to any user worldwide at any time, and EMBL-EBI strongly supports the concept of FAIR data (findable, accessible, interoperable, and resuable) (3). In the case of the European Genome-Phenome Archive (www.ebi.ac.uk/ega), which contains human data consented for research, researchers must request access from a data access committee.

Deposition databases are repositories that archive experimental data on behalf of the entire scientific community and serve as a key component of the scientific record for many data types. These open and searchable resources provide all researchers with direct access to the scientific record, enable access to and re-use of experimental data to verify original results and, by combining multiple data records, provide analytical insights. Deposition databases also provide reference data for the research community and through the use of search tools also allow researchers to rapidly compare their own unpublished data with open access datasets.

Storing experimental data in archival resources is just the first step in extracting knowledge from scientific research. Added-value databases, or knowledgebases, build on archival resources by providing expert curation, annotation, reanalysis, and integration of archived experimental data. Knowledgebases may also provide additional functionality such as searching, analytical tools, data visualization, and linking to related information in other archival resources and knowledgebases. By allowing novel and integrated analysis of archival data, knowledgebases provide an opportunity to reuse data to generate new discoveries. The BioImage Archive (https://www.ebi.ac.uk/bioimage-archive/), introduced below, is a new archival resource for imaging data that will catalyse development of image-related knowledgebases in the future.

## EMBL-EBI DATA RESOURCES: OPEN DATA AND THE GLOBAL DATA INFRASTRUCTURE

EMBL-EBI data resources are open, and their role is to collate, integrate, curate and make freely available to the public the world's scientific data. Adding no constraints on the use and reuse of the data that they serve, our resources provide global access through a multitude of web, programmatic and FTP interfaces, and offer training and user support programmes to facilitate their use. Indeed, an intricate and sophisticated ecosystem of services, tools and data resources exists, not only at EMBL-EBI but also among data resources worldwide, into which open data are immediately—and without human intervention—propagated.

**Figure 1.**
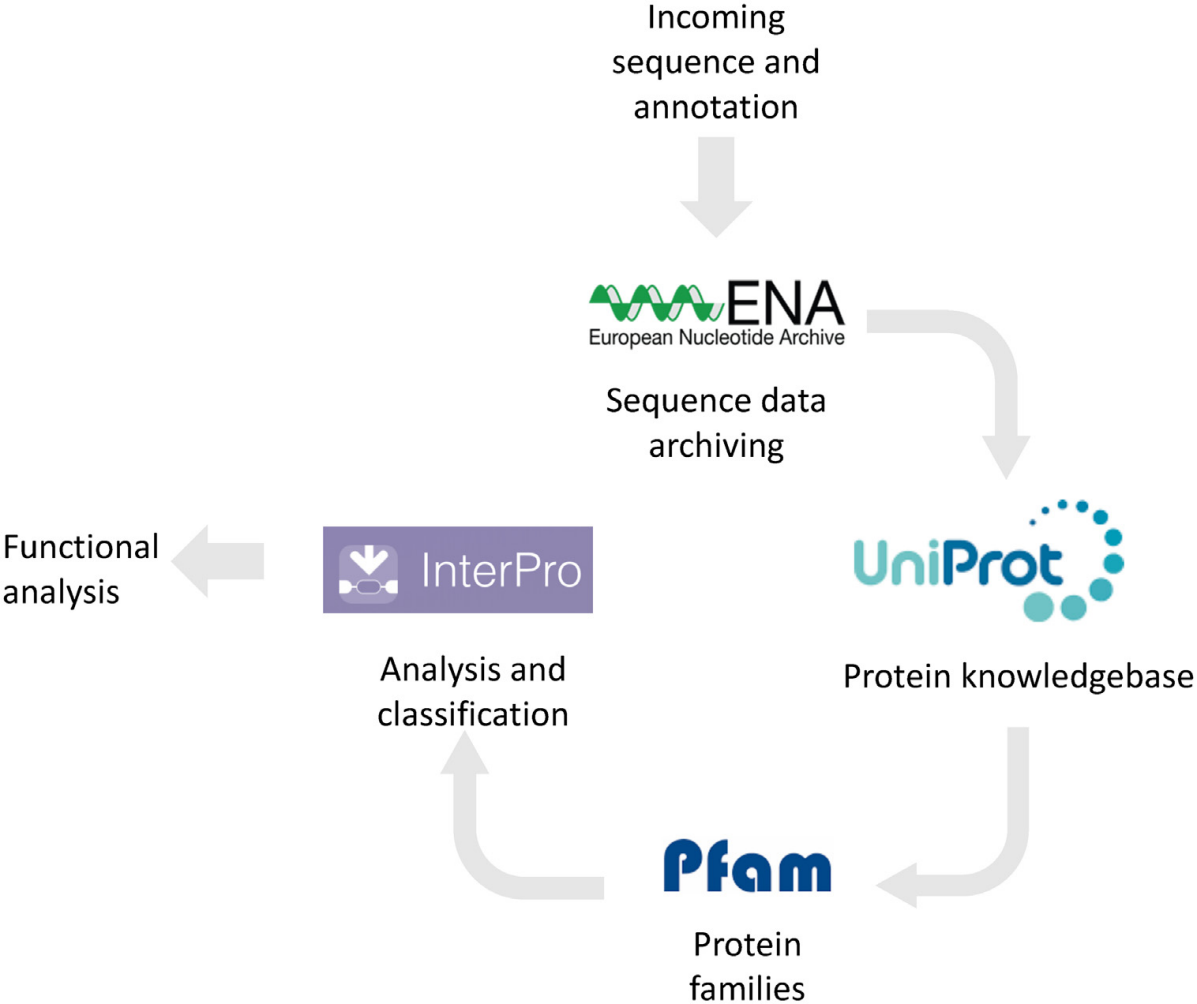
Propagation of open data through the life sciences data infrastructure. An annotated sequence from a newly isolated species autonomously triggers a flow of protein-coding genes into UniProtKB (15), which in turn will propagate data to build sequence family models in Pfam (16) for use in InterPro (17), providing open tools for the functional exploration of further sequences. This example shows only EMBL-EBI resources, but similar data flows occur throughout the entire global infrastructure, as illustrated by the data exchange pathways in Figure 3.

Within EMBL-EBI resources, exchange of data ensures that new information, whether about a gene, protein, structure, or other entity, is shared and searchable across all resources. Data exchange among resources is mediated by application programming interfaces (APIs) (1,4) ensuring that our data resources provide our users with as much information as possible in response to any query. These data exchanges enhance our users’ experience in accessing data and prevent the duplication of effort. Figure 1 provides an example of how new, open data propagates through the EMBL-EBI infrastructure.

**Figure 2.**
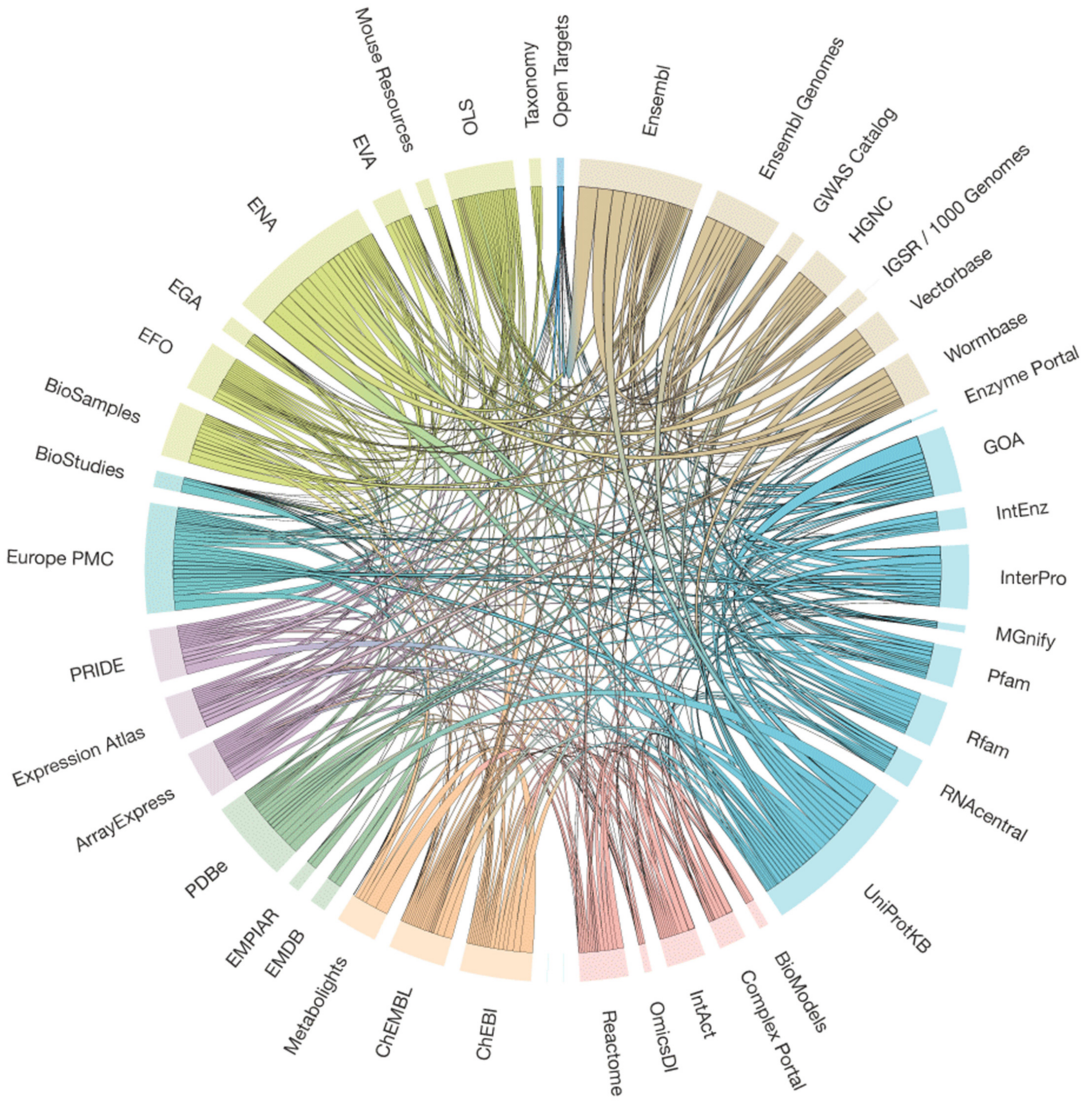
Data exchange between data resources at EMBL-EBI. The dataset contains 911 separate data connections between 41 of EMBL-EBI’s resources. Resources on the circumference of the circle are connected to each other with an internal arc whose width represents the total number of different interactions between the resources. Arc widths are proportional to the number of data connections and do not represent volume of data exchanged. Resources are grouped around the circle by functional cluster and distinguished by colour. Internal arc colours identify each cluster and do not reflect the direction of data exchange. The graphic was generated using the D3 JavaScript library (http://d3js.org) and the dataset was gathered as part of an external review in July 2018.

Figure 2 illustrates data exchange interactions among EMBL-EBI resources. The web of interactions visualised in Figure 2 reflects tremendous effort by the EMBL-EBI teams that develop each resource, and has greatly enhanced the findability and accessibility of data within all resources, increasing their value for all users.

**Figure 3.**
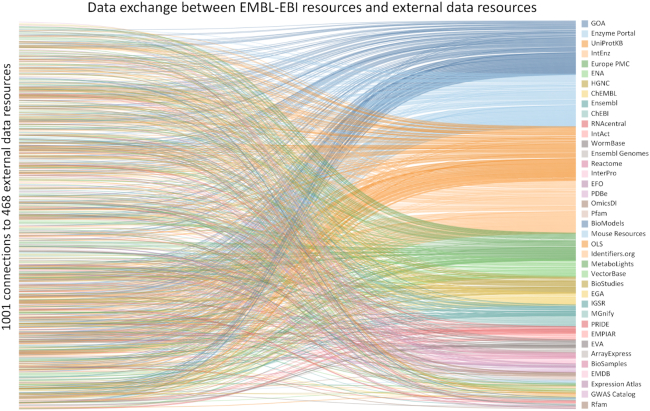
Data exchange between EMBL-EBI resources and external data resources. This Sankey chart shows 468 separate external data resources that are linked to 39 EMBL-EBI resources by 1001 separate data connections. The graphic was generated using Tableau (www.tableau.com) from data gathered as part of an external review in July 2018. For the full dataset, showing all 468 external resources, see supplementary material.

EMBL-EBI resources are not a closed system: together they form a large global infrastructure of data resources that exchange data and information. Most of these data exchanges, just as for exchanges among EMBL-EBI resources, are also implemented through automated protocols using APIs. In principle, we would like to map all of the interactions between life sciences data resources worldwide, but of course this is impractical. As a proxy for the entire worldwide network we have collected interaction data showing exchanges between EMBL-EBI resources and external resources by asking EMBL-EBI data resources to list all known interactions with external resources. These are visualized in Figure 3, which illustrates 1001 different data interactions between 468 external data resources and 39 EMBL-EBI data resources.

It is important to note that this figure does not completely map all data exchanges between EMBL-EBI resources and resources external to EMBL-EBI. EMBL-EBI resources have complete knowledge of incoming data that result from their own requests to outside resources but cannot fully track outgoing data exchanges in which external resources retrieve data from EMBL-EBI resources as such requests are computationally indistinguishable from requests by individual users. EMBL-EBI resources are only aware of outgoing dataflows to other resources when there has been personal communication between external and EMBL-EBI resource teams, for example when multiple resources are members of a consortium; or when the external resource has been in contact with our resource to manage data flows. Figure 3, then, is an undercount, and likely a very large undercount, of the actual data exchanges between EMBL-EBI’s resources and external data resources.

Together, Figures 2 and 3 visually demonstrate the interconnectedness of life sciences data resources and Figure 3 in particular visualises, albeit incompletely, the web of interactions that define the distributed global infrastructure of life sciences data resources. Figure 3 also shows that, while most data exchanges involve other life sciences resources, there are also interactions with some resources, for example Wikipedia, that are broader in scope than just the life sciences. The exchange of data among resources allows individual resources to specialize while ensuring that they can share data with other resources, thus increasing re-use of their data and making it more findable.

## NEW DATA RESOURCES AND FEATURES

### BioImage Archive

Images have been important in the advancement of biology for hundreds of years, but the methods for recording, analyzing, and disseminating them have, of course, evolved from drawing, to printing, to photography, and then to digital methods. Over the last few decades imaging technologies have developed in parallel with advances in molecular biology, as well as computational and data storage capabilities. The newest imaging technologies, for example cryogenic electron microscopy, volume electron microscopy and super-resolution light microscopy, are allowing life-science researchers to observe biological structures and processes in completely new ways.

Imaging technologies represent new opportunities for scientific discovery, but this discovery can be rather difficult. Data volumes, particularly for newer technologies, are high, so sharing and reusing images is challenging. Additionally, imaging is not a single technology, but an umbrella term for data produced by many different methods and representing biological systems at different scales and at different resolutions. Each of these methods also requires new analysis tools, which are best used if they are made available to all researchers.

EMBL-EBI has been collaborating with the wider bioimaging community to address the challenges of storing, accessing, and analyzing imaging data. Pilot projects coordinated by EMBL-EBI, such as EMPIAR (5), as well as collaborations such as the Cell- and Tissue-Image Data Resource (IDR) (6), laid the groundwork (7) for the creation of a public image archive that EMBL-EBI launched in July 2019: the BioImage Archive (https://www.ebi.ac.uk/bioimage-archive/).

**Figure 4.**
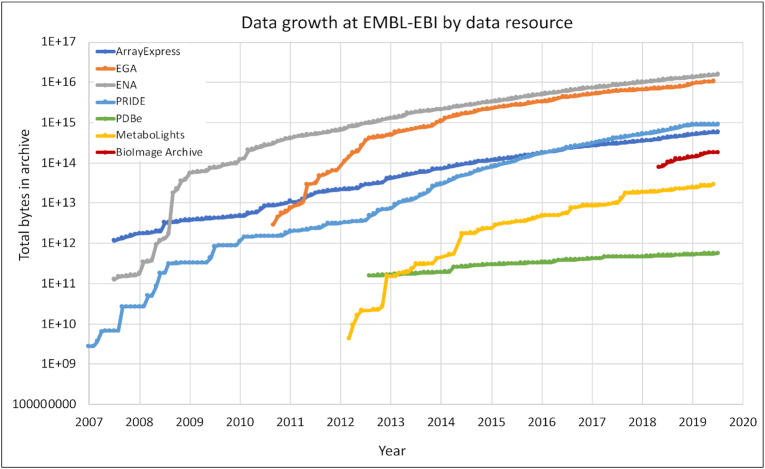
Data accumulation at EMBL-EBI by data resource over time. The y-axis shows total bytes for a single copy of the data resource over time. Resources shown are the BioImage Archive, PRoteomics IDEntifications (PRIDE) (8), European Genome-Phenome Archive (EGA) (14), ArrayExpress (18), European Nucleotide Archive (ENA) (19), Protein Data Bank in Europe (20) and MetaboLights (21). The y-axis for both charts is logarithmic, so not only are most data types growing, but the rate of growth is also increasing. For all data resources shown here growth rates are predicted to continue increasing. The dataset used to generate the figure is available in supplementary material.

The BioImage Archive is a deposition database that stores raw imaging datasets that are either related to a publication or are of value beyond a single experiment, such as large systematic datasets. The archive provides a central data resource that facilitates reproducible research and data reanalysis and accelerates development of image analysis methods. The BioImage Archive will provide data services around which the biological community can develop knowledgebases that add value to archival image data through curation and analysis. Currently, the BioImage Archive accepts electron and x-ray microscopy image data from EMPIAR as well as data from other imaging modalities in the BioStudies resource and the IDR. In Figure 4, we show the size of the BioImage Archive in relation to other large archival resources. The BioImage Archive, while still one to two orders of magnitude smaller than our other large archival resources (e.g. ArrayExpress, EGA, ENA, and PRIDE) is still remarkably large for a new resource. In future, the BioImage Archive will also archive images from other data types so we expect continued growth.

## PRIDE

The PRoteomics IDEntifications database (PRIDE, https://www.ebi.ac.uk/pride/archive/) (8) which is the world-leading proteomics data repository and a member of the ProteomeXchange Consortium (http://www.proteomexchange.org/), has just released a new version that includes a new scalable and fault-tolerant storage backend as well as modern programmatic and web interfaces. This new infrastructure will better support PRIDE users and data submitters, who in 2019 are depositing an average of 310 datasets per month. The activities of PRIDE are increasingly focused on integrating proteomics information into other value-added EMBL-EBI resources. Two recent efforts have accomplished this through integration of phosphorylation data in UniProt (9), and by incorporating cancer-related quantitative proteomics datasets into the Expression Atlas (10). One of PRIDE’s main ongoing projects is to enable a better metadata annotation of submitted datasets in order to facilitate data reuse of public proteomics datasets.

## TRAINING

Training is one of EMBL-EBI’s core missions and a key component of the provision of bioinformatics services to our users (www.ebi.ac.uk/training). External training activities across all parts of EMBL-EBI are coordinated with the goal of creating a coherent, high-quality programme with global reach. The training programme has five major components: courses at EMBL-EBI; courses at host sites; visits and secondments; eLearning (www.ebi.ac.uk/training/online and www.ebi.ac.uk/training/webinars); and train-the-trainer. This combination of activities allows us to deliver training that is both of high quality and scalable.

In 2018, we participated in 355 training and outreach events that supported biomedical and life-science professionals throughout the world; train online was accessed by 485 617 unique IP addresses. We expanded our online training offerings, adding eight new courses and 64 webinars, and we delivered eight train-the-trainer events, training 89 bioinformatics instructors.

We use a competency-based model to design, deliver and measure the impact of our training: we consider which competencies our trainees need to develop, setting appropriate learning objectives, and then following up through surveys to determine whether our trainees have developed and applied these competencies in the longer term (11). We work with a number of consortia to develop competency frameworks for the research community, including the International Society for Computational Biology (12) BioExcel (10.5281/zenodo.264231), CORBEL (10.5281/zenodo.154085) and RItrain (http://ritrain.eu/competency-profile). These projects have helped us to gain a clear appreciation of training needs in several scientific communities, ranging from molecular modellers to research infrastructure providers.

We lead the CABANA project (www.cabana.online), supported by the UK Global Challenges Research Fund, which is addressing the slow implementation of data-driven biology in Latin America by creating a sustainable capacity building programme. CABANA is an international consortium of ten organisations that combines research secondments, workshops, train-the-trainer activities, and new e-learning content to strengthen bioinformatics research in three challenge areas of special relevance to Latin America: communicable disease, sustainable food production and protection of biodiversity. We have completed two successful rounds of selection for our CABANA secondees, who come to the UK for up to six months to work in a bioinformatics group, generally at EMBL-EBI. We have also placed secondees in collaborating institutions including the Earlham Institute and the Roslin Institute. We have a full and varied workshop programme, with training being delivered throughout Latin America, much of it now involving our secondees as instructors. We provide train-the-trainer activities and online training, including live, interactive webinar-based tutorials.

We are constantly developing our training content to keep pace with new disruptive technologies: for example, the UK Medical Research Council recently funded us to develop online and face-to-face training on single-cell RNAseq analysis to enable the research community to make full use of the Human Cell Atlas (https://www.humancellatlas.org/), and through the CABANA consortium we are developing courses on bioinformatics for biodiversity, and on surveillance of communicable disease.

## LOOKING AHEAD

Many of EMBL-EBI’s data resources are deposition databases that receive primary research data. Improvements in technology and decreases in cost continue to drive growth in submissions to the deposition databases and demand on our data services. We continue to manage this growth and demand through technology improvements and by increasing our storage capacity. We also manage growth through participation in collaborations that help manage demand for the data infrastructure through distribution of effort. These collaborations, including for example the International Nucleotide Sequence Database Collaboration (13) and proteomeXchange (8), help manage demand through shared submission and/or storage of research data. EMBL-EBI is also actively supporting in the Global Biodata Coalition, which aids funders in collaborating for more efficient spending on data resources.

Growth in human data is particularly explosive. EMBL-EBI does not directly manage human patient data, but the European Genome-Phenome Archive (EGA) (14) does manage human data that are consented for research. The volume of human data, even the fraction of all human data that are research consented, is now too great for any single institution to manage and the EGA has developed a federated system that distributes storage of datasets while sharing metadata to ease user searching (https://elixir-europe.org/communities/human-data). Such federation is an important approach for sharing the cost and effort of storing large data volumes across multiple institutions and we anticipate federation of other types of data resources in future.

EMBL-EBI will remain heavily engaged in the work of the Global Biodata Coalition global to manage data resources more efficiently, and in the Global Alliance for Genomics and Health (www.ga4gh.org) and its work to create standards to aid exchange and analysis of human and medically-related data across borders and between health systems.

## Supplementary Material

gkz1033_Supplemental_File
